# Psychometric Reliability to Assess the Perception of Women’s Fulfillment of Maternity Rights

**DOI:** 10.3390/ejihpe14080150

**Published:** 2024-08-05

**Authors:** Claudia Susana Silva-Fernández, María de la Calle, Paul Anthony Camacho, Silvia M. Arribas, Eva Garrosa, David Ramiro-Cortijo

**Affiliations:** 1 Department of Biological & Health Psychology, Faculty of Psychology, Universidad Autónoma de Madrid, C/Ivan Pavlov 6, 28049 Madrid, Spain; 2 Obstetric and Gynecology Service, Hospital Universitario La Paz, Paseo de la Castellana 261, 28046 Madrid, Spain; 3 Centro de Investigaciones, Fundación Oftalmológica de Santander, Avenida El Bosque 23, Bucaramanga 680003, Colombia; 4 Department of Physiology, Faculty of Medicine, Universidad Autónoma de Madrid, C/Arzobispo Morcillo 2, 28029 Madrid, Spain; 5 Instituto Universitario de Estudios de la Mujer (IUEM), Universidad Autónoma de Madrid, C/Francisco Tomás y Valiente 5, 28049 Madrid, Spain; 6 Grupo de Investigación en Alimentación, Estrés Oxidativo y Salud Cardiovascular (FOSCH), Instituto de Investigación Sanitaria, Hospital Universitario La Paz (IdiPAZ), 28046 Madrid, Spain

**Keywords:** maternity rights, healthcare during pregnancy, vulnerability, perception of fulfillment

## Abstract

The fulfillment of rights to maternal healthcare is a key factor for the wellbeing of women. However, there is a lack of an instrument to ascertain the experience of women during maternity to enable adequate monitoring. The aim of this study was to validate a new instrument to measure women’s perception of the fulfillment of rights during healthcare in pregnancy and childbirth and immediately postpartum. The initial version of the instrument consists of 50 items and was validated using exploratory factor analysis. Additionally, the final version of the instrument consists of 29 items and was validated by confirmatory factor analysis and known-group validity. The instrument was applied to 185 Spanish women. The global Aiken’s V of the initial instrument proposal was 0.89. The process resulted in an instrument with five factors (information, privacy, consent, support, and participation) that explained the 60% of the total variance. The score of the instrument was correlated with resilience, maternity beliefs, and positive and negative affect. External validation showed relations with age, gravida, and the number of times a woman has been in labor. Additionally, the Cronbach’s α reliability was 0.93 [0.91; 0.94]. In conclusion, the instrument developed is consistent and has appropriate psychometric properties for assessing the fulfillment rights of maternity healthcare.

## 1. Introduction

Maternal health is one of the rights recognized by the United Nations Human Rights Council. The WHO highlights the importance of addressing the mistreatment of women during childbirth to reduce mortality and morbidity in maternal and neonatal care [1]. However, conventional approaches to maternity do not adequately address quality care that are highly valued by mothers. The most frequently described consequences have been unintentional harm, dehumanization, scars, mental illness, and sexual issues [2,3]. Therefore, women continue to experience mistreatment during childbirth [4].

Maternal and neonatal wellbeing are affected by how women are treated during pregnancy and childbirth and postpartum. From European countries, around 20% of women reported abuse during their antenatal care [5]. In addition, in low/middle-income countries, close to 42% of women attending during the postpartum period had experienced physical or verbal abuse or discrimination [6]. Maternity vulnerability is defined as being devoid of care and involving suffering and a loss of value as a human being [7]. The factors that contributed to the experience of vulnerability have been described as younger age, economic difficulties or unemployment, and illiteracy [8]. Among others, the characteristics that could modulate the perception of abuse are negative life events in previous pregnancy or childbirth, and the lack of social support.

The perceived fulfillment of women’s rights during maternity plays a key role in vulnerability during the health care process. An observational study showed that women preferred woman-centered and continuity-of-care models, not only during pregnancy and childbirth but also during the postpartum period [9,10]. The data support the notion that perceived compliance with rights, such as the birth plan, improved clinical maternity outcomes.

The perception of poor quality of healthcare is a powerful determinant of the use of maternity services [11,12]. The universal rights of women and newborns described in the respectful maternity care charter of the White Ribbon Alliance [13] described that everyone has the following rights: (1) to information, informed consent, and for their choices to be respected during maternity care; (2) to privacy and confidentiality; (3) to be treated with dignity and respect; (4) to equality, freedom from discrimination, and equitable care; (5) to the highest attainable level of health; (6) to liberty, autonomy, and self-determination; and (7) for every child to be with their parents or guardians. Therefore, there are international agreements that support all women having access to a humanized maternity care system in which woman-centered care has a positive impact on the birth experiences and outcomes [14]. However, research across countries and cultures needs appropriate instruments that can provide a benchmark to enable meaningful comparisons [3,15,16].

There are instruments focusing on the evaluation of maternal satisfaction with the healthcare received during labor and the hospital stay, mostly evaluated in healthy women with low-risk obstetric pregnancies [17]. The Mothers on Respect (MOR) index is an instrument that assesses the maternity care experience with providers of healthcare options [16]. However, the MOR index does not measure the fulfillment of maternity rights and experiences during the postpartum period. Further research is needed to improve instruments through psychometric testing, considering different maternity periods, and a joint assessment of the perception of the fulfillment of maternity rights and the experience of abuse.

In this article, we describe a women-centered research process to develop and validate a novel instrument to measure the perception of the fulfillment of women’s rights during maternity, from pregnancy to the immediate postpartum period, including the childbirth situation.

## 2. Materials and Methods

### 2.1. Development of the Fulfillment of Maternity Rights Instrument

The fulfillment perception of maternity rights (FMR) is a novel tool that was development as follows. The definition of the construct involved a review of the literature, defining the main variable to be measured, namely women’s perceptions of adequate healthcare during maternity. This first stage led to the publication of a systematic review [3]. Subsequently, the item drafting phase was undertaken, with C.S.S.-F., E.G., and D.R.-C. participating in the brainstorming session to generate the final list of items. The successive stages included judge evaluation and pilot testing, which are described in the following sections. Finally, the FMR was measured based on 50 items.

Based on the universal rights of women and newborns [13,18], the items covering the perception of the women related to adequate health information and requests for informed consent during medical procedures; the privacy and confidentiality of medical data; being treated with dignity and respect; quality healthcare; autonomy; and participation in self-healthcare decisions. The items cover the last pregnancy, childbirth, and the early postpartum (up to 40 days after childbirth). The Spanish version of the original 50-item proposed was showed in Table S1. The scale was ranged from 1 to 4 in a Likert response, in which 0 = “never” and 4 = “always”. The interpretation of the scale would be that a higher score represents a higher perception of the right’s fulfillment. Consistently, the items 33, 41, 48, and 49 were inverted.

### 2.2. Evaluation of the Judges

This evaluation was carried out by five experts in women’s rights and maternity research. The Aiken’s V coefficient (V) was used to analyze the content validity. The possible outcomes ranged from 0 to 1, where 1 represents a perfect agreement among the judges and 0 represents disagreement. The value of this coefficient was considered acceptable at >0.7 [19]. For this study, the language clarity, item relevance, and item coherence were the aspects assessed.

### 2.3. Pilot Test

The comprehensibility of the scale was carried out in 27 women, selected by non-probabilistic sampling at the discretion of the research team. The inclusion criteria of this pilot cohort were women ≥ 18 years; to have undergone labor or a C-section in the last 3 years; to receive healthcare for the last pregnancy, labor, and early postpartum period (up to 40 days after labor); and a good Spanish language understanding. Women were asked to assess the understandability of the questionnaire and to suggest changes if they deemed it appropriate. The women could contact the research team to improve their understanding of the items.

### 2.4. Participants of the Study

Collection and recruitment sampling was performed by social media, an adequate technique for recruitment [20]. During recruitment, 278 women were contacted. Inclusion and exclusion criteria were applied to the women contacted. The inclusion criteria were equal to the pilot cohort. The exclusion criteria were an inability to read/write in Spanish and home birth. Finally, 185 met the inclusion and exclusion criteria. Data were collected from September 2021 to November 2023.

### 2.5. Procedure

A self-administered online tool was prepared by Qualtrics (https://www.qualtrics.com/es/ accessed on 15 July 2021). Firstly, it obtained sociodemographic and obstetric variables and validated psychometric tests. Secondly, it collected the FMR instrument results.

The sociodemographic and obstetric variables were age (years), education level, civil status (single/unmarried vs. any type of relationship), employ status (active working vs. unemployed), parity (number of labor), type of last labor (vaginal vs. C-section), intention of the last pregnancy (yes/no), gestational age of the last pregnancy (weeks), and adverse outcomes (yes/no) during pregnancy (i.e., preeclampsia or gestational diabetes), labor (i.e., premature rupture of the membrane or intrapartum hemorrhage), or early postpartum (such as mastitis or sepsis).

### 2.6. Instruments Used in the Study

To assess divergent validity, women responded to (1) the resilience scale [21], assessing the ability to cope with daily difficulties by 14 items. The higher the score, the greater the woman’s ability to cope with the problems of everyday life. Other studies reported a reliability of 0.88 [22]. (2) The Positive and Negative Affect Schedule (PANAS) [23]. PANAS is used for assessing women’s mood at a specific time by 20 items (10 items measuring positive affect and 10 items measuring negative affect), which are scored based on a 3-point Likert scale from “very low”/“not at all” to “very high”. For the positive score, a higher score indicates more positive emotions. For the negative score, a lower score indicates fewer negative emotions. The PANAS obtained a reliability of 0.87–0.91 [24]. (3) The Maternity Beliefs Scale (MBS), identifying beliefs that women have related to maternity. The MBS has 13 items, clustered in maternity as a sense of life (MBS-life) and maternity as a social duty (MBS-social). The higher the score, the higher the woman’s belief in the domain. The previous reliability was 0.93 [25].

Finally, women responded to the FMR instrument, a tool designed to assess the perception that women have in fulfillment of the maternity rights during pregnancy, labor, or postpartum.

### 2.7. Statistical Analysis

The descriptive analysis was summarized by the mean, median, standard deviation (SD), standard error of mean (SEM), and interquartile range [Q1; Q3], depending on the variable distribution. The skewness and kurtosis were calculated for each item and factor, and the Spearman´s coefficient (Rho) was used to test the correlations.

The data analysis consisted of an exploratory factor analysis (EFA) and was verified by a confirmatory factor analysis (CFA). The EFA was conducted to determine the possible dimensions. The statistic Kaiser–Meyer–Olkin (KMO) and Bartlett’s test of sphericity were used to check if the data were suitable for this analysis. In addition, we followed Cattel´s test and parallel plot, and the number of factors to retain was established, considering the point where the eigenvalue declines steeply and then levels off. A principal component analysis with varimax rotation was used, eliminating items with factor loadings < 0.3 similar to Amado-Mateus [26]. In addition, we also excluded items with complexity (com) > 1.9. Basically, com = 1 indicates that the item loads only on one factor, where a score of 2 evenly loads on two factors.

The factors of the EFA were used for factor conformation. The CFA was estimated by the maximum likelihood. The CFA extracted indexes and thresholds were the minimum discrepancy ratio (χ^2^/df; MR < 5), comparative form index (CFI > 0.9), normed fit index (NFI > 0.9), Tucker–Lewis’s index (TLI > 0.9), adjusted goodness-of-fit index (aGFI > 0.8), and root mean square error of approximation (RMSEA < 0.08). The convergent analysis of the factors was carried out by the Average Variance Extracted (AVE), and the omega (ω) reliability and Cronbach’s alpha (α) were determined. The values considered acceptable were AVE > 0.5, reliability > 0.7, and α > 0.8. Finally, the Maximum Shared Variance (MSV) and Average Shared Variance (ASV) were calculated, the result being acceptable when MSV and ASV are <AVE. The direct score of the factors was calculated by summing the items belonging to the factor and dividing by the number of items in the factor. The standardized (std) factor score was calculated by multiplying the standardized coefficient of CFA of each item and summing each standardized item. The total score (FMR) was calculated by averaging the factor scores. The standardized FMR score was calculated considering the standardized coefficient of the factor in the CFA model.

The validated psychometric scales and subscale scores were standardized by the Rho coefficient, considering a statistical correlation *p*-value (*p*) < 0.05. In addition, an inferential analysis by Mann–Whitney´s U test was conducted to compare groups of women likely to have experienced obstetric vulnerability according to aspects in the literature [3]. A *p*-value (*p*) < 0.05 was considered statistically significant.

The data analyses were performed using R software (version 15.06.2024+R-4.4.1) within the RStudio interface (version 2022.07.1+554, 2022, R Core Team, Vienna, Austria) using the *rio*, *dplyr*, *compareGroups*, *devtools*, *corrplot*, *ggcorrplot*, *psych*, *DiagrammeR,* and *lavaan* packages. For reliability, it was also used the *eRm* and *TAM* packages.

### 2.8. Ethics Statement

This study was approved by the Research Ethics Committees (CEI-112-2199, 22 January 2021). All women willing to participate were given an online information sheet, describing the aims of the study, and the informed consent form was signed in each case. Data collection was anonymous, and databases were blinded. In addition, this study is adhered to the guidelines Standards for the Reporting of Diagnostic Accuracy Studies (STARD) [27] for assessment scale protocols.

## 3. Results

### 3.1. Content Validity

The judges had a high level of agreement for the language clarity (V = 0.90), the item relevance (V = 0.89), and the item coherence (V = 0.89), with the global Aiken’s V coefficient = 0.89. The pilot cohort did not report difficulties understanding any of the items. Therefore, item modifications were not introduced.

### 3.2. Descriptive of Sample

The women were 28.5 ± 0.5 years old; nulliparous women made up 54.6%, and multiparous women 45.4%. Regarding the education level, 5.5% had a primary education, 47.2% a secondary education, and 47.2% university studies. In total, 17.8% of the women were unmarried/single, with 82.2% being married or in a romantic relationship. Related to their employment situation, 55.2% were actively working and 44.8% were unemployed. In 76.7% of the women, the last pregnancy was intended. The gestational age was 38.7 ± 0.1 weeks. In total, 48.5% of the women had a C-section in any gestation. Regarding the obstetric outcomes, pregnancy complications presented in 31.9% women, 17.2% reported complications during labor, and 16.0% had complications during the early postpartum period.

### 3.3. Construct Validity by Factor Analysis

**Exploratory factor analysis:** EFA was performed based on the initial 50 items of the FMR instrument (correlation coefficients are shown in Figure S1). The items showed sphericity (Bartlett’s test: χ^2^ = 9468.3; *p* < 0.001) and the KMO value indicated an acceptable fit (KMO = 0.84). In addition, the parallel analysis suggested we retain seven components. This solution encompassed 56.2% of the explained variance. The items with factor loading values < 0.3 (Items 10, 30, and 41) and complexity values > 1.9 (Items 8, 12, 14, 18, 19, 20, 21, 24, 25, 26, 33, 34, 38, 39, 40, 43, 44, and 48) were excluded (Table S2).

The final solution of the instrument included 29 items. The descriptive analysis of these items is shown in Table 1.

The parallel analysis retained five components, explaining the 60% of the total variance. The Table S3 show the standardized loading based on correlation rotated matrix. According to the EFA, Factor 1 gathers items related to receive adequate healthcare information (Factor 1 = Information), Factor 2 gathers rights related to privacy and confidentiality of health information (Factor 2 = Privacy), Factor 3 refers to consent for the medical procedures (Factor 3 = Consent), Factor 4 refers to social support during maternity (Factor 4 = Support), and Factor 5 was to participation and active listening in medical treatment (Factor 5 = Participation).

**Figure 1:**
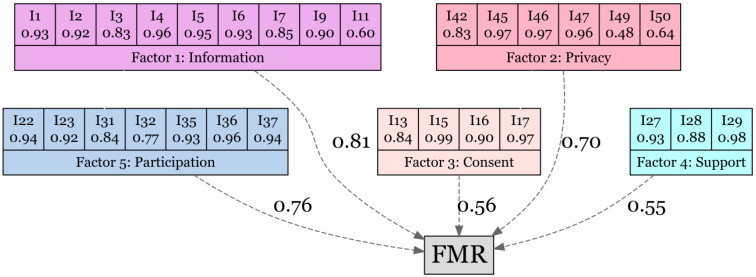
The confirmatory factor analysis model. The value shown represents the standardized estimates variance of each factor and the fulfillment in maternity rights (FMR) global score.

**Confirmatory factor analysis:** CFA was shown by a path diagram (Figure 1) and a descriptive analysis of the factors is reported in Table 2. CFA ensures that data align with expected theoretical constructs, improving the reliability and validity of the FMR measurements. The path diagram visually allows us to identify the relationships between the factors detected by EFA. The value indicates the standardized variance explained by the equations.

The standardized MR of the model was 3.4 (scaled = 2.6). The CFI was 0.99 (scaled = 0.97) and the TLI was 0.99 (scaled = 0.97). The RMSEA was 0.092 [0.085; 0.099], the NFI was 0.66, and the aGFI was 0.55. The global AVE was 0.79 and Cronbach’s α was 0.93 [0.91; 0.94]. All the indexes indicate the factors acceptable consistence. The MSV and ASV were below AVE, indicating the acceptable adequacy of the model. The partial reliability index is shown in Table 3.

**Figure 2:**
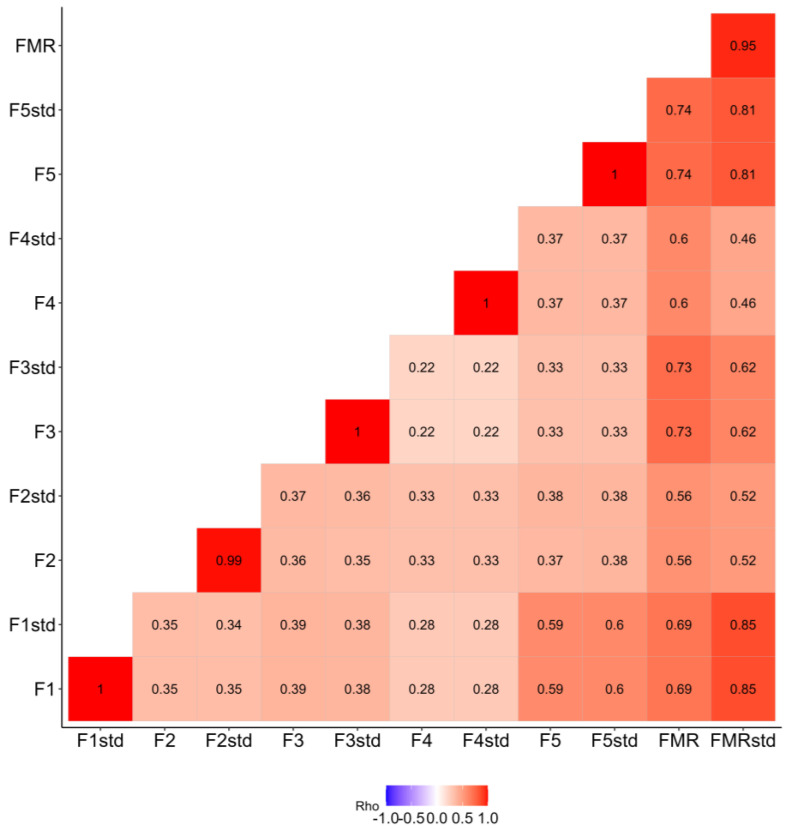
Spearman´s Rho coefficient matrix between factors and the global direct and standardized score of the fulfillment in maternity rights (FMR) instrument. Standardized (std), Factor 1 = Information, Factor 2 = Privacy, Factor 3 = Consent, Factor 4 = Support, Factor 5 = Participation.

The direct score of factors was calculated by averaging the items within the factor. The standardized factor score was obtained by summing the products of the standardized coefficients from CFA. All these scores were positively correlated. Figure 2 shows the correlations between factors and global score of the FMR instrument. Considering the standardized scores were positive and strongly correlated with the direct score, it was considered the direct score for further analysis.

### 3.4. Divergent and Known-Groups Analysis

**Divergent analysis.** Factor 1 (Information) was negatively correlated with the PANAS negative affect (Rho = −0.23; *p* = 0.003) and the MBS-social dimension (Rho = −0.18, *p* = 0.019), and positively with the PANAS positive affect (Rho = 0.23, *p* = 0.003) and resilience (Rho = 0.18, *p* = 0.025). Factor 2 (Privacy) was positively correlated with resilience (Rho = 0.21, *p* = 0.008), and Factor 5 (Participation) and the overall FMR score were positively correlated with the PANAS positive affect (Rho = 0.22, *p* = 0.005; and Rho = 0.20, *p* = 0.010, respectively) and resilience (Rho = 0.27, *p* < 0.001; and Rho = 0.21, *p* = 0.010, respectively). Factors 3 (Consent) and 4 (Support) were not significantly shown to correlate.

**Known-groups analysis.** The factors did not show statistical significance with the type of labor and complications during labor. However, Factor 1 (Information) was significantly lower in women with non-intended pregnancy than in those with an intended pregnancy. Factor 4 (Support), Factor 5 (Participation), and the overall FMR were significantly lower in women who had postpartum complications compared to women who did not develop these complications (Table 4). In addition, Factor 1 was positively correlated with women’s age (Rho = 0.16, *p* = 0.027), gravida (Rho = 0.15, *p* = 0.034), and the number of labors (Rho = 0.20, *p* = 0.005). Factor 5 (Rho = 0.17, *p* = 0.022) and FMR (Rho = 0.15, *p* = 0.044) were also positively and significantly correlated with the number of labors.

The final version of the FMR instrument, ordered by factor and stage of maternity, is shown in the Appendix A.

## 4. Discussion

International conventions protect women against mistreatment during maternity healthcare services [4]. However, to suffer abuse during maternity care is still a globally emerging issue. The FMR instrument is suitable to measure women’s perceptions of the maternity process encompassing pregnancy, childbirth, and the immediate postpartum period. The FMR measures the rights to adequate health information, privacy and confidentiality, autonomy, and participation, and social support. Previous data showed the relevance of integrating measures into an instrument measuring the fulfillment of healthcare in maternity rights and in different maternal periods [3,12]. The FMR instrument is consistent and has appropriate psychometric properties, having been evaluated in a specific sociocultural and health context. The FMR instrument can have similarities with the MOR index, which measures women’s experiences when interacting with primary maternity care providers [16]. However, FMR assesses the compliance with general maternity rights and assesses a broader period from pregnancy to the postpartum period. Furthermore, FMR evaluates not only the interaction with health providers but also the relationship with the health institution and women´s social support. Nevertheless, both scales could be complementary, since the validation of the MOR was performed in broader sociocultural contexts with good psychometric properties.

In most cases, the women had stable emotional support when the last pregnancy was intentional. The gestational age exceeded 37 completed weeks and, overall, the pregnancy, labor, or postpartum adverse outcomes did not exceed 35%. Additionally, the cohort was balanced between parity, educational level, employment status, and the type of the last delivery (vaginal vs. C-section). It is important to consider these characteristics since the perception of the fulfillment of rights may be influenced by the social context [8]. It would be interesting to validate the FMR instrument in other contexts to be able to compare scores.

For the psychometry evaluation, the EFA revealed items that should be excluded from the original proposed as they may jeopardize the construct validity. These items showed a low factor loading, indicating that none of them were associated with the latent variable [28]. In addition, the complexity represents the number of latent variables needed to account for the observed items. Items with complexity values close to two imply that their variance may be distributed in two or more factors [29]. These items were excluded to avoid a situation where several dimensions were explained simultaneously for the same item. For example, the original Item I8 (“*You had sufficient and clear information about the medical procedures performed during childbirth*”) was excluded because Item I2 (“*You had sufficient and clear information about the healthcare procedures that you and your newborn should have for an effective delivery/C-section*”) already covered the latent variable. Thus, the final FMR instrument was designed to comprise 29 items covering the dimensions of the respectful maternity care charter [13], the right to be informed and request consent, and have their choices respected during maternity care; the right to privacy and confidentiality; and the right to request the social support necessary during maternity. All items follow the women-centered proposal.

According to the CFA fit indices, the absolute indices (aGFI) and relative index (NFI) did not pass the cut-off. However, other relative indices (CFI and TLI) showed that the 5-factors model was acceptable [30]. In general, the FMR instrument presented a good reliability. All the factors had positive and significant correlations between them. The validation showed that both direct and standardized scores can be used. However, we recommend using the direct scores, as they have been the most thoroughly validated in this article.

Moreover, the FMR scores correlated with critical psychological variables during maternity. In Chinese women, it was demonstrated that positive affect had a positive correlation with maternal role adaptation, with negative affect having the opposite trend [31]. In addition, women could adjust to positive emotions, but they were unable to regulate negative ones [32,33]. Subsequently, these feelings tended to evolve as the pregnancy progresses and the perception of it changed. Although there are data relating PANAS scores to maternal wellbeing [34], few studies associate these psychological components with maternity rights compliance. Our data highlight that increases in FMR scores were correlated with higher levels of positive emotions during motherhood. Furthermore, the fatigue of the women after labor was negatively associated with her positive affect [34]. Additionally, resilience during pregnancy can be modulated the association between trait anger in gestation and postnatal depression [35]. According to our data, resilience and positive affect correlated positively with a better perceived fulfillment of rights, but particularly with the right to information, privacy, and participation in maternity healthcare decisions. It must be considered that motherhood changes the social role of women. This change can alter life experiences and the perception of an environment that demands more than it offers [36]. Thus, it would be useful to evaluate what feelings women have about the experience of maternity as a criterion to be met by society. In addition, for maternal adaptation to society, the information and active role of women in the care process are relevant factors [10,25,37]. Our data support that the perception of the fulfillment of the right to adequate information increased when the negative affect or belief in motherhood as a social duty were low. Conceding the bidirectional association between better women’s mental health and better social relationships [38], it is important to promote real and optimal social support during all stages of maternity.

As mentioned above, the analysis of the social context in the fulfillment of rights should be central. The observational data showed that the expectations and experiences of childbirth vary by maternal age [39]. The FMR instrument supports that older woman and those with previous pregnancies expressed greater compliance with information rights. Women around their 30s have shown an increase in life satisfaction from pregnancy to postpartum [40], with the stability of the result depending on the sociocultural context [41]. Women with advanced maternal age (<35 years) have a higher perception of pregnancy risk than younger woman, regardless of their medical risk [42]. It seems that to have first newborn older has psychological advantages over younger counterparts, reporting lower symptoms of depression and anxiety during pregnancy [43]. This implies that life experiences are key in the assessment of perceptions. Similarly, having had previous pregnancies and labors provided a higher perception of information rights compliance, participation in medical decision making, and overall FMR.

It was demonstrated that the feelings of the women related to inadequate healthcare provision during pregnancy, childbirth, and postpartum were the source of their problems [44]. Many of the women reported that they had not been taken seriously by health care providers, and some felt seriously neglected [44]. In Italian nulliparous women, it was revealed that the more fear of childbirth, the worse their maternity experience, with no significant effect on C-sections [45]. In other observational study, it was reported that abuse in healthcare was associated with a fear of childbirth (aOR = 2.25 in nulliparous and aOR = 4.04 in multiparous women) [5]. Therefore, special attention should be paid to the postpartum period since hormonal adjustment and adaptation to the role of motherhood in society can be decisive in the perception of rights violations. In line with our data, the women with lower scores on the fulfillment of support rights, participation, and overall FMR were those with postpartum complications. This shows that FMR scores are linked to the woman’s postpartum unpleasant experiences. The women considered that childbirth and postpartum are decisive situations to perceive motherhood as procedures in which their rights may be vulnerated.

Another important aspect is the intentionality of the pregnancy. When the pregnancy is unplanned, it may demand major adjustments that exceed the woman’s coping [46]. Thus, the FMR instrument is also sensitive to this aspect. Perceived compliance with the right to adequate health information was lower among women who had an unintended pregnancy. Further research would be necessary to determine the counseling policies and supportive care for women experiencing an unplanned pregnancy.

This psychometric study is an important advance that contributes to the research and monitoring of good practices in maternity care. It also responds to the WHO’s call for attention to the mistreatment of women during childbirth and promotes the improvement of maternal services. The psychometric analysis was rigorous, but the FMR was validated in a Spanish sociocultural context. It would be necessary to know the health background of women to interpret the outcomes. Additionally, the enrollment process was performed via social media, which may affect the supervision of responses and identity verification. In-person recruitment allows for interviewer–participant interaction and the collection of latent variables that can influence scores. Other potential limitations could be the sample size, which was smaller than in other psychometric studies. It highlights the need to prioritize respectful, woman-centered care during pregnancy, childbirth, and postpartum. The findings underscore the importance of ensuring access to clear and adequate information, as well as respect for women’s privacy and autonomy at all stages of maternal care. The current data also highlight the importance of providing adequate social support to women during this critical period. These findings support the need for health policies and clinical practices that promote respectful, informed maternity care focused on women’s individual needs.

## 5. Conclusions

The fulfillment in maternity rights instrument has exhibited strong psychometric properties for assessing maternal perceptions on healthcare rights. The instrument assesses dimensions such as adequate health information, the right to privacy and confidentiality of medical data, the right to autonomy and participation in your own healthcare decisions, and social support during the maternity process. Cultural and health context is needed to clarify the complexity of the scores. Health institutions can use this instrument to assess and adapt maternity protocols for women users to perceive that all their human rights are covered, moving towards more and more humanized models.

## Figures and Tables

**Table 1:** Descriptive analysis of the final items included in the FMR instrument.

Item	Mean	SD	SEM	Median	Q1	Q3	Range	Skew	Kurtosis
I1	3.19	1.28	0.09	4.00	3.00	4.00	4	−1.37	0.42
I2	2.96	1.41	0.10	4.00	2.00	4.00	4	−1.05	−0.40
I3	2.90	1.38	0.10	4.00	2.00	4.00	4	−0.90	−0.66
I4	2.91	1.42	0.10	4.00	2.00	4.00	4	−1.01	−0.46
I5	2.81	1.46	0.11	3.00	2.00	4.00	4	−0.87	−0.76
I6	2.72	1.47	0.11	3.00	1.00	4.00	4	−0.74	−0.97
I7	3.23	1.28	0.09	4.00	3.00	4.00	4	−1.50	0.78
I9	3.06	1.37	0.10	4.00	3.00	4.00	4	−1.21	−0.03
I11	2.97	1.42	0.10	4.00	2.00	4.00	4	−1.05	−0.44
I13	3.03	1.41	0.10	4.00	2.00	4.00	4	−1.12	−0.27
I15	2.57	1.69	0.12	4.00	1.00	4.00	4	−0.58	−1.43
I16	2.63	1.67	0.12	4.00	1.00	4.00	4	−0.63	−1.37
I17	2.48	1.74	0.13	3.00	0.00	4.00	4	−0.50	−1.55
I22	3.48	1.04	0.08	4.00	3.00	4.00	4	−2.02	3.05
I23	3.48	1.01	0.07	4.00	3.00	4.00	4	−2.02	3.19
I27	3.06	1.48	0.11	4.00	2.00	4.00	4	−1.15	−0.37
I28	3.08	1.51	0.11	4.00	3.00	4.00	4	−1.23	−0.25
I29	3.27	1.31	0.10	4.00	3.00	4.00	4	−1.51	0.71
I31	3.01	1.45	0.11	4.00	2.00	4.00	4	−1.10	−0.40
I32	3.11	1.39	0.10	4.00	3.00	4.00	4	−1.28	0.09
I35	2.95	1.38	0.10	4.00	2.00	4.00	4	−1.04	−0.37
I36	2.75	1.49	0.11	3.00	1.00	4.00	4	−0.78	−0.95
I37	2.78	1.49	0.11	3.00	2.00	4.00	4	−0.84	−0.85
I42	3.58	1.00	0.07	4.00	4.00	4.00	4	−2.67	6.23
I45	3.73	0.75	0.05	4.00	4.00	4.00	4	−3.35	11.57
I46	3.66	0.88	0.06	4.00	4.00	4.00	4	−3.03	8.71
I47	3.68	0.83	0.06	4.00	4.00	4.00	4	−3.12	9.77
I49	3.66	0.92	0.07	4.00	4.00	4.00	4	−3.02	8.52
I50	3.63	0.99	0.07	4.00	4.00	4.00	4	−2.72	6.30

Standard deviation (SD); Standard error of mean (SEM); Quartile (Q).

**Table 2:** Descriptive analysis of the factor and global scores (direct and standardized) of the FMR instrument.

	Mean	SD	SEM	Median	Q1	Q3	Range	Skew	Kurtosis
Factor 1	2.97	1.09	0.08	3.22	2.56	3.89	4.00	−1.12	0.30
Factor 2	3.66	0.66	0.05	4.00	3.50	4.00	4.00	−3.26	12.22
Factor 3	2.68	1.41	0.10	3.00	1.00	4.00	4.00	−0.57	−1.23
Factor 4	3.14	1.28	0.09	4.00	2.33	4.00	4.00	−1.24	0.16
Factor 5	3.08	0.96	0.07	3.43	2.43	4.00	4.00	−0.98	0.30
FMR	15.53	3.86	0.28	16.48	13.52	18.55	17.67	−1.13	0.82
Factor 1 std	23.37	8.71	0.64	25.96	20.12	30.88	31.48	−1.11	0.23
Factor 2 std	17.76	3.33	0.24	19.40	17.48	19.40	19.40	−3.28	11.91
Factor 3 std	9.86	5.27	0.39	11.32	3.83	14.80	14.80	−0.56	−1.26
Factor 4 std	8.76	3.57	0.26	11.16	6.61	11.16	11.16	−1.25	0.18
Factor 5 std	19.34	6.06	0.45	21.52	14.76	25.12	25.12	−0.97	0.28
FMR std	56.40	14.41	1.06	61.37	49.18	66.39	63.53	−1.19	0.79

Standardized (std); Standard deviation (SD); Standard error of mean (SEM); Quartile (Q). Factor 1 = Information, Factor 2 = Privacy, Factor 3 = Consent, Factor 4 = Support, Factor 5 = Participation.

**Table 3:** Internal consistence of factors and FMR instrument.

	Factor 1 Information	Factor 2 Privacy	Factor 3 Consent	Factor 4 Support	Factor 5 Participation	FMR
AVE	0.78	0.69	0.86	0.87	0.81	0.79
MSV	0.41	0.17	0.12	0.12	0.41	-
ASV	0.57	0.50	0.72	0.70	0.49	-
Omega	0.95	0.86	0.94	0.90	0.94	0.97
Alpha	0.95	0.90	0.93	0.95	0.90	0.93

Average variance extracted (AVE); Maximum shared variance (MSV); Average shared variance (ASV); Omega (ω) reliability; Cronbach’s alpha (α).

**Table 4:** External validation by known groups of factors and global score of the FRM instrument.

	Non-Intended Pregnancy	Intended Pregnancy	*p*	Vaginal	C-Section	*p*
Factor 1	2.89 [1.56; 3.33]	3.33 [2.64; 4.00]	0.048	3.33 [2.67; 3.89]	3.11 [2.44; 3.89]	0.350
Factor 2	3.83 [3.33; 4.00]	4.00 [3.67; 4.00]	0.117	3.83 [3.50; 4.00]	4.00 [3.67; 4.00]	0.113
Factor 3	3.00 [1.75; 4.00]	3.25 [1.00; 4.00]	0.523	3.00 [1.00; 4.00]	3.00 [2.00; 4.00]	0.395
Factor 4	4.00 [3.00; 4.00]	4.00 [2.58; 4.00]	0.408	3.00 [1.00; 4.00]	4.00 [2.42; 4.00]	0.460
Factor 5	3.29 [2.14; 3.71]	3.50 [2.39; 4.00]	0.250	3.43 [2.43; 4.00]	3.29 [2.29; 4.00]	0.961
FMR	16.0 [12.1; 17.2]	16.7 [13.8; 18.7]	0.157	16.4 [13.1; 18.5]	16.7 [13.8; 18.6]	0.682
	**Uncomplicated** **pregnancy**	**Complicated** **pregnancy**	*p*	**Uncomplicated** **postpartum**	**Complicated** **postpartum**	***p***
Factor 1	3.33 [2.67; 4.00]	3.22 [2.25; 3.78]	0.340	3.33 [2.67; 4.00]	2.89 [2.11; 3.67]	0.103
Factor 2	4.00 [3.50; 4.00]	4.00 [3.50; 4.00]	0.654	4.00 [3.50; 4.00]	3.83 [3.50; 4.00]	0.238
Factor 3	3.00 [1.00; 4.00]	3.12 [2.00; 4.00]	0.429	3.50 [1.25; 4.00]	2.25 [1.00; 3.75]	0.084
Factor 4	4.00 [2.33; 4.00]	4.00 [2.42; 4.00]	0.774	4.00 [2.92; 4.00]	3.00 [2.00; 4.00]	0.012
Factor 5	3.57 [2.43; 4.00]	3.07 [2.29; 3.71]	0.078	3.57 [2.43; 4.00]	2.86 [2.14; 3.57]	0.002
FMR	16.6 [13.8; 18.7]	16.4 [13.3; 18.1]	0.505	16.7 [13.9; 18.7]	14.7 [12.7; 17.1]	0.005
	**Uncomplicated** **pregnancy**	**Complicated** **pregnancy**	*p*			
Factor 1	3.22 [2.50; 4.00]	3.17 [2.67; 3.67]	0.539			
Factor 2	4.00 [3.50; 4.00]	4.00 [3.50; 4.00]	0.739			
Factor 3	3.00 [1.00; 4.00]	3.12 [1.44; 4.00]	0.968			
Factor 4	4.00 [2.33; 4.00]	4.00 [2.25; 4.00]	0.605			
Factor 5	3.43 [2.43; 4.00]	3.07 [2.32; 3.71]	0.495			
FMR	16.6 [13.5; 18.6]	16.4 [14.1; 18.1]	0.618			

Data show the median and interquartile range [Q1; Q3]. The *p*-value (*p*) was extracted from Mann–Whitney´s U test. Factor 1 = Information, Factor 2 = Privacy, Factor 3 = Consent, Factor 4 = Support, Factor 5 = Participation.

## Data Availability

The data of this study are available upon request from the corresponding author. The availability of the data is restricted to investigators based in academic institutions.

## Supplementary Materials

The following supporting information can be downloaded at: https://www.mdpi.com/article/10.3390/ejihpe14080150/s1, Figure S1: The corplot by Spearman’s rho coefficient of the original items of the FMR instrument; Table S1: The original items of the fulfillment in maternity rights instrument and exclusion criteria by exploratory factor analysis and principal component analysis; Table S2: Factor loading and complexity of the original FMR instrument; Table S3: Standardized factor loading, communality, and uniqueness extracted from a rotated matrix of the final version of the FMR instrument.

## Appendix A

The fulfillment in maternity rights scale (FMR) is as follows.

Instructions: The following describes your perception related to maternity rights in health services. Following the response scale, mark with an “X” how often you perceived that the following situations occurred about the healthcare process during your last pregnancy, labor, and immediately postpartum (up to 40 days after labor).

0 = Never/1 = Sometimes/2 = Half of the time/3 = Often/4 = Always.

**Table:**

**Item**	**Factor**	**During Last Pregnancy**	**0**	**1**	**2**	**3**	**4**
I1	1	You had sufficient and clear information about the healthcare procedures that you and your newborn should have for a healthy pregnancy.					
I4	1	You had sufficient and clear information about the administrative steps you had to take to ensure your healthcare.					
I7	1	You had sufficient and clear information about the medical procedures performed on you.					
I42	2	Your privacy or avoidance of exposure of your body (covering intimate parts, regulating the exhibition of your body to persons not involved in the treatment) was respected during healthcare.					
I45	2	Confidentiality of information about your health was maintained during healthcare.					
I15	3	Your written consent was requested for healthcare procedures involving the invasion of your body (vaginal examinations or ultrasound).					
I27	4	If you requested family support or accompaniment during healthcare, they were allowed access. *If you never requested it, check option “4”.*					
I35	5	During the healthcare, your feelings, emotions, doubts, and opinions were listened to.					
**Item**	**Factor**	**During last labor**	**0**	**1**	**2**	**3**	**4**
I2	1	You had sufficient and clear information about the healthcare procedures that you and your newborn should have for an effective delivery/C-section.					
I5	1	You had sufficient and clear information about the administrative steps you had to take to ensure your healthcare.					
I46	2	Confidentiality of information about your and your newborn’s health status was maintained during healthcare.					
I50	2	If your rights were violated during healthcare, you consider that you defended them. *If your rights were not violated, check option “4”.*					
I13	3	You were asked for your verbal consent for healthcare processes that involved the invasion of your body (perineal cutting or ultrasounds).					
I16	3	You were asked for your written consent for healthcare processes involving the invasion of your body (perineal cutting or ultrasound).					
I28	4	If you requested family support or accompaniment during healthcare, they were allowed access. *If you never requested it, check option “4”.*					
I22	5	They respected your decisions on self-care and newborn care behaviors that were not risky for either of you.					
I31	5	Health professionals performed or allowed you to perform pain control (medication, application of compresses, walking, postural control).					
I36	5	During healthcare, they listened to your feelings, emotions, doubts, and opinions.					
**Item**	**Factor**	**During last immediate postpartum (up to 40 days after labor)**	**0**	**1**	**2**	**3**	**4**
I3	1	You had sufficient and clear information about the healthcare procedures that you and your newborn should have for a healthy postpartum period.					
I6	1	You had sufficient and clear information about the administrative steps you had to take to ensure your healthcare.					
I9	1	You had sufficient and clear information about the medical procedures that were performed on you.					
I11	1	You had sufficient and clear information to initiate and maintain breastfeeding in a correct and painless way.					
I47	2	Confidentiality of information about your and your newborn’s health status was maintained during healthcare.					
I49	2	You consider that some of the healthcare practices you received may have been both uncomfortable and unnecessary (application of bandages, among others).					
I17	3	You were asked for your written consent for healthcare procedures that involved invasion of your body (dressings, tubal ligation, or ultrasound).					
I29	4	In the case of having requested family support or accompaniment during healthcare, they were you allowed access. *If you never requested it, check option “4”.*					
I23	5	They respected your decisions on self-care and newborn care behaviors that were not risky for either of you (type of contraception, type of breastfeeding, among others).					
I32	5	Health professionals performed or allowed to you to perform actions to control pain (medication, application of compresses, etc.).					
I37	5	During healthcare, they listened to your feelings, emotions, doubts, and opinions.					

Factor 1 gathers items related to receive adequate healthcare information (Factor 1 = Information), Factor 2 gathers rights related to privacy and confidentiality of health information (Factor 2 = Privacy), Factor 3 refers to consent to medical procedures (Factor 3 = Consent), Factor 4 refers to social support during maternity (Factor 4 = Support), and Factor 5 to participation and active listening in medical treatment (Factor 5 = Participation).
