# Supplementary material for: Psychometric Reliability to Assess the Perception of Women’s Fulfillment of Maternity Rights
Source: Eur J Investig Health Psychol Educ. 2024 Aug 5;14(8):2248–61. doi: 10.3390/ejihpe14080150 (PMC11353840; doi:10.3390/ejihpe14080150)
Supplement: Supplementary file 1 [file ejihpe-14-00150-s001.zip › ejihpe-3121175-supplementary.pdf]

Table S1. The original items of fulfillment in maternity rights instrument and exclusion criteria by exploratory factor analysis and principal component analysis.

**Item**	**Sentence**	**Motive to be excluded**
**I1**	Tuvo información suficiente y clara sobre los cuidados y procedimientos de salud que debía tener hacía usted y el recién nacido, para un embarazo sano y eficaz *Durante el embarazo*	
**I2**	Tuvo información suficiente y clara sobre los cuidados y procedimientos de salud que debía tener hacía usted y el recién nacido, para un parto/cesárea eficaz *Durante el parto*	
**I3**	Tuvo información suficiente y clara sobre los cuidados y procedimientos de salud que debía tener hacía usted y el recién nacido, para un posparto sano *Durante el postparto*	
**I4**	Tuvo información suficiente y clara sobre las gestiones administrativas que usted debía hacer para garantizar su atención médica *Durante el embarazo*	
**I5**	Tuvo información suficiente y clara sobre las gestiones administrativas que usted debía hacer para garantizar su atención médica *Durante el parto*	
**I6**	Tuvo información suficiente y clara sobre las gestiones administrativas que usted debía hacer para garantizar su atención médica *Durante el postparto*	
**I7**	Tuvo información suficiente y clara sobre los procedimientos médicos que le realizaron *Durante el embarazo*	
**I8**	Tuvo información suficiente y clara sobre los procedimientos médicos que le realizaron *Durante el parto*	Initial EFA. Complexity >1.9
**I9**	Tuvo información suficiente y clara sobre los procedimientos médicos que le realizaron *Durante el postparto*	
**I10**	Los profesionales de salud le brindaron información clara y suficiente sobre los métodos de anticoncepción (quirúrgicos, de barrera u hormonales) disponibles para su planificación familiar *Durante el embarazo y/o posparto*	Initial PCA. Factor loading <0.3
**I11**	Tuvo información suficiente y clara para iniciar y mantener la lactancia materna de forma correcta y no dolorosa *Durante el postparto*	
**I12**	Le solicitaron su consentimiento/autorización verbal para los procesos de atención médica que implicaban la invasión de su cuerpo (tactos vaginales o ecografías) *Durante el embarazo*	Initial EFA. Complexity >1.9
**I13**	Le solicitaron su consentimiento/autorización verbal para los procesos de atención médica que implicaban la invasión de su cuerpo (corte en el perineo o ecografías) *Durante el parto*	
**I14**	Le solicitaron su consentimiento/autorización verbal para los procesos de atención médica que implicaban la invasión de su cuerpo (curas, ligadura de trompas o ecografías) *Durante el postparto*	Initial EFA. Complexity >1.9
**I15**	Le solicitaron su consentimiento/autorización escrita para los procesos de atención médica que implicaban la invasión de su cuerpo (tactos vaginales o ecografías) *Durante el embarazo*	
**I16**	Le solicitaron su consentimiento/autorización escrita para los procesos de atención clínica que implicaban la invasión de su cuerpo (corte en el perineo o ecografías) *Durante el parto*	
**I17**	Le solicitaron su consentimiento/autorización escrita para los procesos de atención clínica que implicaban la invasión de su cuerpo (curas, ligadura de trompas o ecografías) *Durante el postparto*	
**I18**	Respetaron sus decisiones sobre el curso de su tratamiento clínico (la fecha de programación de cesárea, entre otras) *Durante el embarazo*	Initial EFA. Complexity >1.9
**I19**	Respetaron sus decisiones sobre el curso de su tratamiento clínico (aceleración del parto, el tipo de parto –vaginal o cesárea, entre otras) *Durante el parto*	Initial EFA. Complexity >1.9
**I20**	Respetaron sus decisiones sobre el curso de su tratamiento clínico (ligadura de trompas, entre otras) *Durante el postparto*	Initial EFA. Complexity >1.9
**I21**	Respetaron sus decisiones en conductas de cuidado propio y del recién nacido que no eran de riesgo para ninguno de los dos *Durante el embarazo*	Initial EFA. Complexity >1.9

**I22**	Respetaron sus decisiones en conductas de cuidado propio y del recién nacido que no eran de riesgo para ninguno de los dos *Durante el parto*	
**I23**	Respetaron sus decisiones en conductas de cuidado propio y del recién nacido que no eran de riesgo para ninguno de los dos (tipo de anticoncepción, tipo de lactancia -materna o de fórmula-, entre otros) *Durante el postparto*	
**I24**	Los profesionales e instituciones de salud solicitaron y brindaron los procesos, recursos y servicios de atención médica necesarios para garantizar su salud física y mental, y la de su recién nacido (remitir a otros profesionales, o tratamientos necesarios para su salud) *Durante el embarazo*	Initial EFA. Complexity >1.9
**I25**	Los profesionales e instituciones de salud solicitaron y brindaron los procesos, recursos y servicios de atención médica necesarios para garantizar su salud física y mental, y la de su recién nacido *Durante el parto*	Initial EFA. Complexity >1.9
**I26**	Los profesionales e instituciones de salud solicitaron y brindaron los procesos, recursos y servicios de atención médica necesarios para garantizar su salud física y mental, y la de su recién nacido (remitir a otros profesionales o tratamientos necesarios para su salud) *Durante el postparto*	Initial EFA. Complexity >1.9
**I27**	En el caso de haber solicitado el apoyo familiar o acompañamiento durante la atención médica, le permitieron el acceso a su acompañante. *Si nunca lo solicitó marque la opción «4».* *Durante el embarazo*	
**I28**	En el caso de haber solicitado el apoyo familiar o acompañamiento durante la atención médica, le permitieron el acceso a su acompañante. *Si nunca lo solicitó marque la opción «4».* *Durante el parto*	
**I29**	En el caso de haber solicitado el apoyo familiar o acompañamiento durante la atención médica, le permitieron el acceso a su acompañante. *Si nunca lo solicitó marque la opción «4».* *Durante el postparto*	
**I30**	Los profesionales de salud realizaron o le permitieron acciones para el control de su dolor (medicación, entre otras) *Durante el embarazo*	Initial PCA. Factor loading <0.3
**I31**	Los profesionales de salud realizaron o le permitieron acciones para el control de su dolor (medicación, aplicación de compresas, o admitirle deambular, control postural) *Durante el parto*	
**I32**	Los profesionales de salud realizaron o le permitieron acciones para el control de su dolor (medicación, aplicación de compresas, entre otros) *Durante el postparto*	
**I33**	Los profesionales que le atendieron le maltrataron físicamente *Durante el parto*	Initial EFA. Complexity >1.9
**I34**	En la atención médica, fueron protegidos sus derechos (como el derecho a la vida, la libertad, la salud y protección social-económica-cultural, la igualdad, entre otros) *Durante el postparto*	Initial EFA. Complexity >1.9
**I35**	Durante la atención médica escucharon sus sentimientos, emociones, dudas y opiniones *Durante el embarazo*	
**I36**	Durante la atención médica escucharon sus sentimientos, emociones, dudas y opiniones *Durante el parto*	
**I37**	Durante la atención médica escucharon sus sentimientos, emociones, dudas y opiniones *Durante el postparto*	
**I38**	Las personas que le atendieron mantuvieron una comunicación respetuosa y motivadora hacia usted y su maternidad *Durante el embarazo*	Initial EFA. Complexity >1.9
**I39**	Las personas que le atendieron mantuvieron una comunicación respetuosa y motivadora hacia usted y su maternidad *Durante el parto*	Initial EFA. Complexity >1.9
**I40**	Las personas que le atendieron mantuvieron una comunicación respetuosa y motivadora hacia usted y su maternidad *Durante el postparto*	Initial EFA. Complexity >1.9
**I41**	Si presentó dificultades para pagar los servicios de salud en una hospitalización, fue retenida por la institución hasta realizar el pago completo. *De no haber tenido dificultad para el pago o no haber estado hospitalizada, seleccione la opción «4».* *Durante el parto*	Initial PCA. Factor loading <0.3
**I42**	Su privacidad o evitar la exposición de su cuerpo (cubrir partes íntimas, regular la exhibición de su cuerpo a persona no implicadas en el tratamiento), fue respetada durante la atención médica *Durante el embarazo*	

**I43**	Su privacidad o evitar la exposición de su cuerpo (cubrir partes íntimas, regular la exhibición de su cuerpo a persona no implicadas en el tratamiento), fue respetada durante la atención médica *Durante el parto*	Initial EFA. Complexity >1.9
**I44**	Su privacidad o evitar la exposición de su cuerpo (cubrir partes íntimas, regular la exhibición de su cuerpo a persona no implicadas en el tratamiento), fue respetada durante la atención médica *Durante el postparto*	Initial EFA. Complexity >1.9
**I45**	La confidencialidad de la información sobre su estado de salud fue conservada durante la atención médica *Durante el embarazo*	
**I46**	La confidencialidad de la información sobre su estado de salud y el de su recién nacido, fue conservada durante la atención médica *Durante el parto*	
**I47**	La confidencialidad de la información sobre su estado de salud y el de su recién nacido, fue conservada durante la atención médica *Durante el postparto*	
**I48**	Considera que alguna práctica de atención médica que le realizaron pudo ser molesta e innecesaria a la vez (aplicación de intervenciones -subirse sobre el abdomen para empujar al recién nacido hacia el canal del parto-, entre otras) *Durante el parto*	Initial EFA. Complexity >1.9
**I49**	Considera que alguna práctica de atención médica que le realizaron pudo ser molesta e innecesaria a la vez (aplicación de curas, entre otras) *Durante el postparto*	
**I50**	Si sus derechos fueron vulnerados durante la atención médica, considera que los defendió. *En el caso que sus derechos no hayan sido vulnerados, responda la opción «4». Durante el parto*	

Figure S1. The corplot by Spearman´s rho coefficient of the original items of FMR instrument.

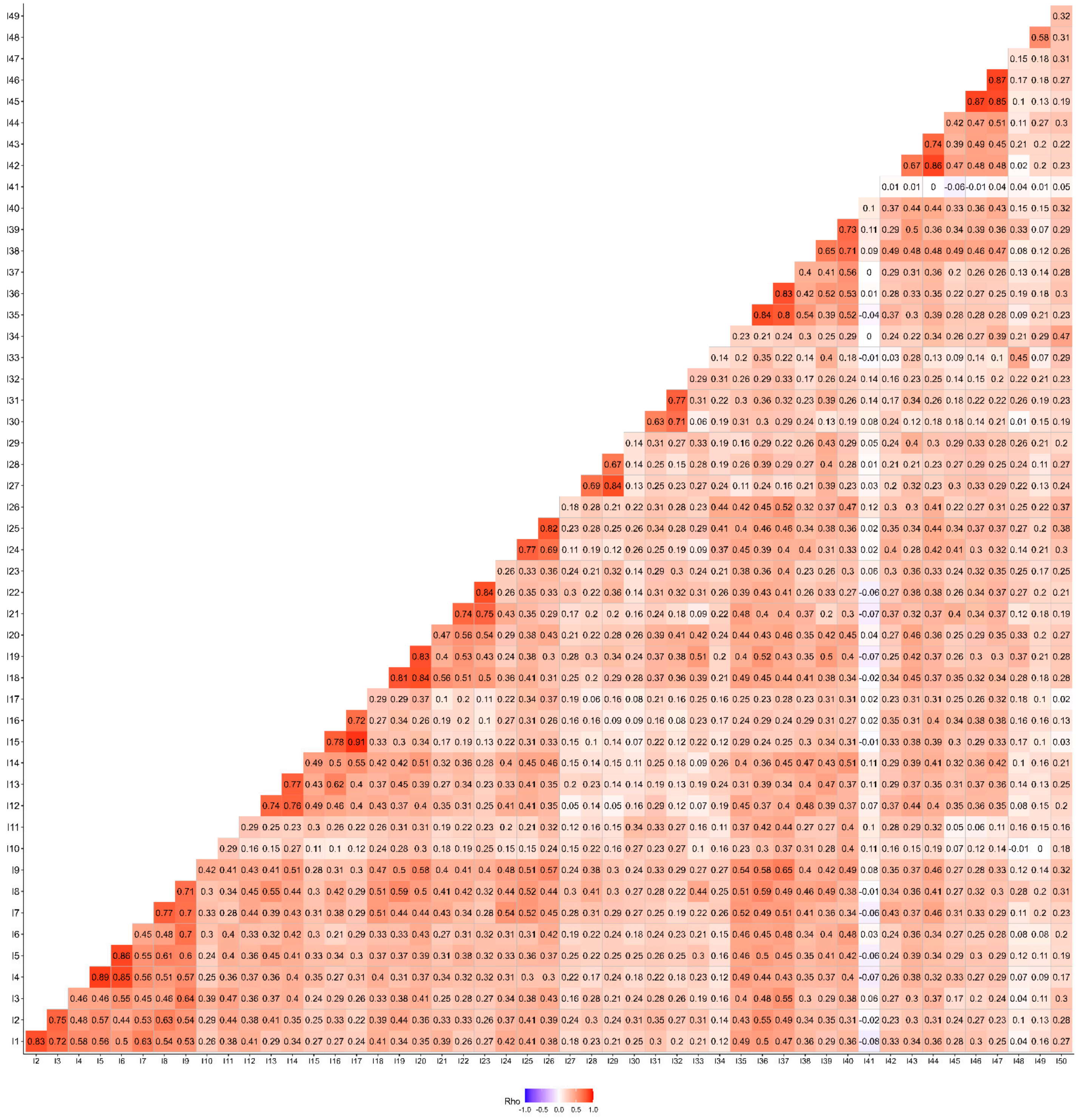

Table S2. Factor loading and complexity of the original of FMR instrument.

Item	Factor 1	Factor 2	Factor 3	Factor 4	Factor 5	Factor 6	Factor 7	Com	Exclusion
**I1**	0.25	0.52	0.20	0.35				1.3	
**I2**	0.17	0.45	0.35	0.34			0.10	1.4	
**I3**	0.17	0.44	0.26	0.33				1.3	
**I4**	0.17	0.91		0.16	0.13			1.4	
**I5**	0.13	0.88	0.17	0.17	0.11		0.14	1.3	
**I6**	0.14	0.87		0.20		0.12		1.2	
**I7**	0.28	0.50	0.33	0.38	0.13			1.8	
**I8**	0.24	0.45	0.48	0.36			0.24	2.4	2
**I9**	0.23	0.56	0.33	0.39		0.13	0.12	1.6	
**I10**		0.22	0.21	0.20				1.6	1
**I11**		0.32	0.23	0.33	0.15			1.6	
**I12**	0.26	0.16		0.21	0.31		0.66	2.2	2
**I13**	0.21	0.19	0.19	0.13	0.22		0.90	1.9	
**I14**	0.27	0.23		0.19	0.28	0.13	0.65	2.3	2
**I15**	0.17	0.13	0.15		0.95		0.13	1.2	
**I16**	0.22	0.11	0.20		0.70		0.35	1.2	
**I17**	0.14	0.14	0.19		0.87		0.13	1.2	
**I18**	0.28	0.24	0.48	0.33	0.14	0.20		3.3	2
**I19**	0.20	0.19	0.67	0.28	0.10	0.12	0.19	4.2	2
**I20**	0.20	0.23	0.57	0.25	0.20	0.25		3.3	2
**I21**	0.27	0.13		0.21		0.70		2.1	2
**I22**	0.14	0.11	0.39	0.11		0.80	0.12	1.5	
**I23**	0.17	0.10	0.28	0.13		0.89		1.5	
**I24**	0.48	0.17	0.11	0.37			0.14	3.6	2
**I25**	0.42	0.14	0.29	0.36	0.14		0.14	2.8	2
**I26**	0.32	0.19	0.20	0.40	0.21			3.0	2
**I27**	0.17	0.15	0.48			0.14		1.3	
**I28**	0.18	0.13	0.38	0.17				1.6	
**I29**	0.20	0.16	0.53			0.21		1.4	
**I30**	0.17	0.14	0.25	0.28				1.4	1
**I31**	0.16		0.41	0.25	0.13	0.10		1.6	
**I32**	0.13		0.43	0.18		0.14		1.4	
**I33**	-0.13	0.16	0.65		0.16			5.1	2
**I34**	0.35		0.16	0.12				2.5	2
**I35**	0.18	0.34		0.81		0.23		1.8	
**I36**	0.12	0.29	0.24	0.80		0.16	0.14	1.9	
**I37**	0.13	0.28	0.17	0.80		0.17		1.8	
**I38**	0.48	0.32	0.13	0.33	0.13		0.14	3.1	2
**I39**	0.28	0.26	0.43	0.29	0.14		0.19	4.7	2
**I40**	0.36	0.33	0.16	0.41	0.15			3.0	2
**I41**							0.13	3.4	1
**I42**	0.70	0.13	0.11	0.14	0.13	0.21		1.5	
**I43**	0.45	0.19	0.33		0.14	0.27	0.11	3.7	2
**I44**	0.65	0.18	0.22	0.15	0.15	0.26		2.0	2
**I45**	0.90	0.16			0.11	0.11		1.5	
**I46**	0.84	0.17	0.12		0.13			1.7	
**I47**	0.85	0.12	0.18		0.12	0.21		1.8	

**I48**	0.15		0.54					2.0	2
**I49**	0.32		0.27					1.8	
**I50**	0.33		0.26	0.25	-0.14			1.7	

Com: complexity; Exclusion criteria 1: factor loading<0.3; Exclusion criteria 2: Initial complexity>1.9.

Table S3. Standardized factor loading, communality and uniqueness extracted from rotated matrix of the final version of FMR instrument.

Item	Factor 1	Factor 2	Factor 3	Factor 4	Factor 5	Communality	Uniqueness
**I1**	0.70	0.22	0.07	0.04	0.24	0.60	0.40
**I2**	0.63	0.15	0.10	0.11	0.31	0.54	0.46
**I3**	0.63	0.13	0.08	0.08	0.28	0.51	0.49
**I4**	0.85	0.17	0.14	0.10	-0.02	0.78	0.22
**I5**	0.85	0.13	0.17	0.16	0.04	0.79	0.21
**I6**	0.83	0.14	0.09	0.11	0.04	0.73	0.27
**I7**	0.63	0.24	0.21	0.17	0.24	0.58	0.42
**I9**	0.68	0.20	0.13	0.21	0.32	0.67	0.33
**I11**	0.42	-0.03	0.15	0.07	0.33	0.31	0.69
**I13**	0.28	0.22	0.42	0.11	0.16	0.34	0.66
**I15**	0.15	0.12	0.94	0.05	0.09	0.93	0.07
**I16**	0.17	0.20	0.81	0.05	0.07	0.74	0.26
**I17**	0.15	0.11	0.88	0.07	0.10	0.83	0.17
**I22**	0.12	0.27	0.03	0.32	0.50	0.44	0.56
**I23**	0.12	0.29	-0.03	0.28	0.48	0.41	0.59
**I27**	0.11	0.12	0.13	0.86	0.12	0.80	0.20
**I28**	0.20	0.12	0.03	0.62	0.18	0.47	0.53
**I29**	0.13	0.17	0.05	0.85	0.21	0.82	0.18
**I31**	0.12	0.12	0.15	0.20	0.49	0.34	0.66
**I32**	0.08	0.12	0.09	0.19	0.47	0.29	0.71
**I35**	0.54	0.17	0.08	-0.06	0.59	0.67	0.33
**I36**	0.51	0.10	0.10	0.06	0.65	0.71	0.29
**I37**	0.51	0.10	0.10	-0.02	0.64	0.70	0.30
**I42**	0.21	0.63	0.15	0.14	0.18	0.52	0.48
**I45**	0.18	0.91	0.15	0.10	0.06	0.89	0.11
**I46**	0.16	0.83	0.19	0.14	0.02	0.78	0.22
**I47**	0.15	0.88	0.18	0.13	0.18	0.87	0.13
**I49**	0.07	0.32	0.07	0.03	0.18	0.15	0.85
**I50**	0.16	0.32	-0.07	0.03	0.23	0.19	0.81
